# 17.1 A RANDOMIZED CONTROLLED TRIAL OF CANNABIDIOL IN SCHIZOPHRENIA

**DOI:** 10.1093/schbul/sby014.065

**Published:** 2018-04-01

**Authors:** Philip McGuire, Philip Robson, Wiesław Cubała, Daniel Vasile, Paul Morrison, Rachel Barron, Adam Taylor, Stephen Wright

**Affiliations:** 1 Institute of Psychiatry, Psychology & Neuroscience, King’s College London; 2 GW Pharmaceuticals; 3 Medical University of Gdańsk; 4 Central Military Emergency University Hospital, Bucharest

## Abstract

**Background:**

Both preclinical and human research suggest that cannabidiol (CBD) has antipsychotic properties. This study assessed the safety and effectiveness of CBD in patients with schizophrenia.

**Methods:**

Patients with schizophrenia (n=88) were randomized to receive CBD (1000 mg/day) or placebo alongside their existing antipsychotic medication for 6 weeks. Participants were assessed before and after treatment using the PANSS, BACS, GAF scales, and the CGI Improvement and Severity scales.

**Results:**

Compared those given placebo, patients treated with CBD had lower levels of positive psychotic symptoms (PANSS; p=0.02), and were more likely to have been rated by clinicians as improved (CGI-I; p=0.02) and as not severely unwell (CGI-S; p=0.04). Patients who received CBD also showed trends for greater improvements in cognitive performance (BACS; p=0.07) and in overall functioning (GAF; p=0.08). There was no difference in the frequency of CBD of adverse events between CBD and placebo.

**Discussion:**

These data suggest that CBD has beneficial effects in patients with schizophrenia and is not associated with significant adverse effects.

